# Assessment of the effects of a work-related allergy to seafood on the reduction of earning capacity in the context of BK No. 5101 

**DOI:** 10.5414/AL0DB380E

**Published:** 2021-01-14

**Authors:** Heinrich Dickel, Annette Kuehn, Beate Dickel, Andrea Bauer, Detlef Becker, Manigé Fartasch, Michael Haeberle, Swen Malte John, Vera Mahler, Christoph Skudlik, Elke Weisshaar, Thomas Werfel, Johannes Geier, Thomas Ludwig Diepgen

**Affiliations:** 1 Department of Dermatology, Venerology and Allergology, St. Josef-Hospital, University Hospital of the Ruhr University Bochum (UK RUB), Bochum, Germany,; 2 Department of Infection and Immunity, Luxembourg Institute of Health, Esch-sur-Alzette, Luxembourg,; 3 Dermatological Practice Dr. med. Peter Wenzel, Hattingen, Germany,; 4 Department of Dermatology, University Allergy Center, University Hospital Carl Gustav Carus, Technical University, Dresden, Germany,; 5 Department of Dermatology, University Medical Center, Mainz, Germany,; 6 Institute for Prevention and Occupational Medicine (IPA) of the German Social Accident Insurance, Department of Clinical and Experimental Occupational Dermatology, Ruhr University Bochum, Bochum, Germany,; 7 Dermatological Practice, Künzelsau, Germany,; 8 Department of Dermatology, Environmental Medicine and Health Theory, University of Osnabrück and Institute for Interdisciplinary Dermatological Prevention and Rehabilitation (iDerm) at the University of Osnabrück, Osnabrück, Germany,; 9 Paul-Ehrlich-Institut (PEI), Langen, Germany,; 10 Division of Occupational Dermatology, Department of Dermatology, University Hospital Heidelberg, Heidelberg, Germany,; 11 Department of Dermatology and Allergy, Hannover Medical School, Hannover, Germany,; 12 Information Network of Departments of Dermatology (IVDK), Institute at the University Medical Center Göttingen, Göttingen, Germany,; 13 University of Heidelberg, Heidelberg, Germany; † The authors dedicate the present work to Prof. Dr. med. Thomas Ludwig Diepgen who founded the working group “Evaluation of Allergens with regard to BK No. 5101” and who was a committed leader of this group until his much too early death.

**Keywords:** immediate-type allergy, occupational dermatology, BK No. 5101, fish, crustaceans, shellfish, seafood, reduction of earning capacity, mollusks

## Abstract

Fish, crustaceans, and mollusks are among the most potent allergenic foods of animal origin and are thus important triggers of work-related immediate-food allergies. In Germany, work-related seafood allergies are of great importance in the fishing and processing industries as well as in the areas of food preparation, food control, and food sales. There is no causal therapy of seafood allergy, only the strict and lifelong avoidance of allergens remains. The following recommendations serve to assess the impact of a seafood allergy with regard to the work opportunities ended by it for the assessment of the reduction of earning capacity (MdE (German for Minderung der Erwerbsfähigkeit)) in the context of the occupational disease number 5101 of the Annex to the German regulation for occupational diseases. As a special feature of work-related seafood allergy with regard to insurance law aspects, it must be taken into account that there is a potential risk of systemic reaction with subsequent multi-organ involvement. For the estimation of MdE in the general labor market, the impact of a seafood allergy can therefore be assessed, depending on its clinical severity, as generally “mild” to “severe” in justified individual cases.

**German version published in Dermatologie in Beruf und Umwelt. Vol. 68, No. 3/2020, pp. 113-125. DOI 10.5414/DBX00380.**

## Background 

The edible seafoods fish and shellfish are regarded as extremely high-quality and particularly digestible foods [86, 87] containing easily assimilable proteins, essential polyunsaturated omega-3 fatty acids (e.g., eicosapentaenoic acid, docosahexaenoic acid) as well as a large number of important vitamins (e.g., vitamin D), minerals, and trace elements (e.g., iodine, iron, selenium, zinc) [11]. Fish is divided into saltwater fish (62% market share in Germany in 2018; https://www.fischinfo.de/index.php/markt/92-datenfakten/4979-marktanteile-2019, last access: 11/07/2020) and freshwater fish (26% market share in 2018). In Germany, the most popular edible fish include pollack, salmon, tuna, herring, and trout. Shellfish (12% market share 2018) includes crustaceans and mollusks [29]. Commonly eaten crustaceans include shrimp, crawfish, crayfish, and lobster [42] while the most popular mollusks are mussels, octopus, and squid [53]. 

Seafood, however, is considered to be the most potent allergenic food of animal origin [11, 87], and thus regarded as one of the most common triggers of food allergies [6, 87, 100]. In addition, seafood is also an important trigger of occupational allergies [12, 87, 100]. The most widely studied seafood allergies are those based on an IgE-mediated mechanism [42]. Parvalbumins, enolases, aldolases, and collagens are allergens of primary importance in fish allergies [40, 57]. For shellfish allergies, tropomyosins, arginine kinases, sarcoplasmic calcium binding proteins, myosin light chains, troponin C, and triose phosphate isomerases have been described as important allergens [57, 67]. Clinical cross-reactions between fish and shellfish are not known [40]. 

An important aspect of seafood allergies is that clinical symptoms may occur not only during consumption but also during food processing and preparation. In the context of professional activities, the impact can be significant for the affected person, leading to allergic reactions so severe that the person has to give up work [12]. Until now, there has been no framework for the assessment of the “impact of an allergy” for occupational seafood allergies [12], respective cases could only be assessed in the declaratory procedure by analogy with the work-related immediate-type allergy to natural rubber latex [77], because proteins with the potential risk of anaphylaxis and systemic disease also play a significant role in natural rubber latex. 

## Epidemiology 

The prevalence of seafood allergies varies greatly depending on country-specific eating habits, the size of the seafood processing industries, and the density of gastronomy [42, 53]. Countries with a high proportion of coastal regions, such as the Mediterranean countries, Scandinavia, and Japan, are particularly affected [59, 63, 86, 87]. In general, shellfish allergies seem to be more common than fish allergies [10, 29]. For the adult population in Europe, prevalence (based on self-assessment/sensitization/medical history and sensitization/provocation testing) of up to 1.5% (95% confidence interval (CI) 1.0 – 2.2%)/2.9% (95% CI 2.2 – 3.9%)/0.8% (95% CI 0.2 – 2.5%)/0.2% (95% CI 0 – 0.9%) for fish allergies and up to 2.0% (95% CI 1.2 – 3.3%)/10.3% (95% CI 7.0 – 14.9%)/0.2% (95% CI 0.1 – 0.5%)/0.3% (95% CI 0.1 – 1.0%) for shellfish allergies have been reported [58]. 

In the fish- and shellfish-processing industry, the prevalence of occupational allergic contact urticaria or protein contact dermatitis ranges from 3 to 11% worldwide [34], that of work-related allergic rhinitis from 5 to 24% [33, 52], and that of work-related allergic asthma from 2 to 36% [21, 31, 33]. Occupational allergic asthma seems to be more often associated with shellfish (4 – 36%) than with fish (2 – 8%) [33]. 

## Clinical findings 

Seafood contains extremely potent IgE-reactive allergens that can cause mild to moderate reactions but also life-threatening anaphylaxis upon skin contact, inhalation, or ingestion [18, 49, 55, 100]. These are immediate-type reactions characterized by common manifestation within the first 2 hours after exposure [19, 50, 54, 67]. Biologically active seafood allergens could be detected in sera of healthy subjects only 10 minutes after ingestion [91]. Late reactions, of up to 8 hours after ingestion, have also been reported [67]. 

The clinical reactions are manifold. They can affect single organs but also several organ systems [40, 50, 54, 83]. They frequently occur in combination on the skin and mucous membranes of the respiratory and gastrointestinal tract, ranging from contact urticaria and protein contact dermatitis to the oral allergy syndrome (itching, angioedema, dysphagia) [12, 15], upper (rhinitis, conjunctivitis, dyspnea) and lower respiratory symptoms (asthma) [5, 32, 52] as well as gastrointestinal (nausea, vomiting, cramps, diarrhea) [54] and circulatory problems up to the life-threatening or lethal-ending anaphylactic shock [7, 11, 12, 18, 55, 67, 84, 85, 86, 87]. 

Allergic skin reactions are usually triggered by direct contact [54]. Both the oral allergy syndrome and upper respiratory tract symptoms are commonly caused by ingestion and inhalation [12]. Upper respiratory symptoms can be seen as an early risk marker for the pathogenesis of allergic asthma [32, 33, 34, 52], which might develop after weeks, months, or several years [32, 64]. Severe to life-threatening anaphylaxis is usually observed after ingestion [12, 18, 50, 55, 59, 75]. However, skin reactions – especially generalized urticaria – can occur in addition to the direct contact in the context of a systemic allergic reaction after ingestion or inhalation [12, 26, 28, 32, 34]. On the other hand, in the case of highly sensitized individuals, direct skin contact alone might induce systemic reactions [12, 26, 32, 34]. Individuals suffering from isolated contact urticaria after exclusive skin contact with seafood, who may develop a generalized urticaria over time [26], are exceptional cases, and so far, have only been observed in cooks and fishmongers [12, 20, 61]. 

In the occupational environment, irritant hand eczema often manifests first, co-triggered and maintained by a high moisture exposure and simultaneous contact with primarily skin-irritating seafood components (e.g., fluids with enzymatic activity of trypsin and pepsin), often based on an atopic skin diathesis [12, 13, 25, 32, 96, 97]. With such an impaired skin barrier, an allergic contact urticaria in the sense of a “two-phase eczema” can develop [71] and with increasing chronicity, an allergic protein contact dermatitis – the latter being the second most frequent occupational dermatosis in patients with occupational food contact [96]. 

However, IgE-mediated reactions are not the only cause of seafood intolerances [10, 67, 86, 87]. Adverse effects can also be caused by toxic compounds, bacterial spoiling, or pharmacological effects. The latter are mostly due to biogenic amines and, of these, especially histamine [30], which is only present in small amounts in fresh seafood. Under microbial influence, histamine can be produced in large quantities [40]. In addition, seafood allergies can also be mimicked by reactions to food additives or to parasites, especially to the parasitic nematode *Anisakis simplex* [10, 40, 59, 82, 100]. 

## Seafood allergens and sensitization profiles 

### Fish allergens 

Parvalbumin (10 – 12 kDa) is the main allergen in fish muscles [27, 29, 40, 42, 67]. More than 70% of fish allergic patients are sensitized to this major allergen [24, 49, 50, 75]. The parvalbumin content varies considerably between different fish species and thus influences their allergenicity [48, 84, 87]. Herring and carp, for example, have a high parvalbumin content of up to 5 mg allergen/g muscle tissue, whereas tuna is virtually free of this allergen (≈ 0.03 mg/g muscle tissue) [48, 86]. All parvalbumins are characterized by an extraordinary molecular stability against heat, denaturing agents, and proteolytic enzymes [27, 43, 49, 84, 85, 87, 89]. This high stability as well as their high content in many fish species is probably the reason why even low amounts of fish can be sufficient to trigger allergic reactions [87, 100]. Enolases (50 kDa) and aldolases (40 kDa) are also allergenic muscle proteins. They have been isolated from salmon, tuna, and cod [27, 29, 45, 87]. Their stability is significantly lower as compared to parvalbumins [40, 67]. Collagens (330 kDa) have been identified as further fish allergens [27, 40, 67]. IgE-reactivity has been shown for both fish collagen and fish gelatin, a hydrolyzed collagen product [41, 44, 45]. The correlation between anti-collagen IgE-reactivity and clinical reactions has been best described in Japanese patients [39]. Vitellogenin (118 kDa) is only found in fish roe, but at high levels [82]. Beyond parvalbumin, up to one third of the fish allergic patients are sensitized to the minor allergens described above for individual fish species [29, 45, 49, 75, 82, 87, 92]. 

### Shellfish allergens 

Tropomyosin is the main allergen in shellfish species [24, 29, 42, 50, 53, 54, 67]. It is a 65 – 70 kDa water-soluble muscle protein. Arginine kinases (minor allergens) are 40 – 45 kDa muscle proteins that play a key role in the energy production of invertebrates and therefore occur in significant amounts in crustaceans and mollusks [29, 53, 67]. Homologous allergens in house dust mites, cockroaches, and flour moths are known [10, 17, 54]. Tropomyosin, the low-molecular minor allergens myosin light chains (17 – 20 kDa) and sarcoplasmic calcium-binding proteins (20 – 25 kDa) are heat-stable [17, 29, 53, 67]. 

Other proteins that are vital for the muscle function were purified as crustaceans’ allergens, troponin C and I (21 and 30 kDa), and triose phosphate isomerase (28 kDa) [17, 29, 53, 67]. Finally, hemocyanin (75 kDa), the blood pigment of crustaceans, has been described as an allergen in the context of clinical anaphylaxis to shrimp [17, 29, 67]. 

### Immunological cross-reactions 

Cross-reactions between fish and shellfish allergens are not known so far [11, 17, 27, 40, 42, 54]. Consequently, allergic persons do not necessarily have to avoid all types of seafood [63]. According to epidemiological studies [90, 94], ca. 20 – 40% of patients suffer from co-allergies to fish and shellfish [86]. 

Relevant cross-reactions among different fish species are well known [29, 67, 86], with different phenotypes being observed. Most fish allergic patients react to a broad range of different fish species [40]. Parvalbumins are of great importance as cross-reactive allergens [87], as they are characterized by a high protein sequence homology (60 – 80%) and structural similarity [54, 85] – prominent examples are Gad c 1 from cod and Cyp c 1 from carp. Cross-reactions between minor allergens of different fish species (e.g., enolase, aldolase, or collagen) may also occur. Importantly, other patients might react to only a few or even only to single fish species [16, 29, 40, 46, 67]. This points to both the existence of additional fish species-specific lgE-binding epitopes on parvalbumins and other distinct allergy triggers [86, 87]. Furthermore, parvalbumins, enolases, and aldolases have been identified as cross-reactive allergens in fish/chicken meat (so-called “fish-chicken syndrome”) [11, 27, 41, 47, 87]. Parvalbumins from frog and crocodile meat are also known as cross-reactive allergens [27, 40, 87]. 

Due to the high sequence homologies among crustacean tropomyosins (82 – 100%) and among mollusk homologs (65 – 99%) [50, 53, 67], commonly observed cross-reactions can be explained in shellfish allergic patients [17, 29, 54, 76, 100]. The cross-reactivity rate of shellfish tropomyosins (75%) is significantly higher than the cross-reactivity rate of fish parvalbumins (50%) [10, 67, 75, 89]. However, since the sequence homology between crustacean and mollusk tropomyosins is only 50 – 60%, crustacean-sensitized patients are not necessarily reactive to mollusks and vice versa [24, 32, 67]. In patients with shellfish allergies, cross-reactions to mites and cockroaches have also been described, the so-called “mite-shellfish syndrome”, and explained by the involvement of cross-reactive tropomyosins [17, 29, 33, 53, 67, 100]. Also, de-novo sensitizations to shrimp allergens have been reported in patients under specific immunotherapy for house dust mite allergy [93]. A homologous tropomyosin allergen (Ani s 3) has been discovered in the parasitic nematode *Anisakis simplex*. This parasite resides in the crustacean muscle, and even more commonly in the fish muscle, and thus can be ingested together with the infested host animal [82, 100]. 

### Sensitization 

Allergies to seafood often develop in childhood, although the prevalence is higher in adulthood [29, 86, 87]. Sensitization occurs mainly via the gastrointestinal tract through ingestion [29, 54, 67, 75]. Secondary sensitization to shellfish seems to be due to cross-reactivity in the context of the “mite-shellfish syndrome”, meaning via primary sensitization to mite and cockroach allergens occurring through respiratory exposure [24, 33, 54, 55, 89]. 

Primary sensitization in the workplace is mainly via the respiratory tract by inhalation of aerosolized seafood particles (e.g., vapors from boiling or drying seafood) [29, 34, 52, 67, 84, 87]. Finally, sensitization may also be caused by direct skin contact, for example in cases of pre-existing skin barrier damage [35], and/or fish preparation without protective gloves [4, 26, 28, 29, 34, 67, 71, 84]. 

### Special remarks on seafood allergenicity 

Quite a number of allergic persons report clinical symptoms to seafood exclusively depending on its method of preparation [4]. Cooking, frying, grilling, salting, drying, or freezing can reduce or increase the allergenicity to seafood. For example, fish that is kept on ice for several days seems to contain intact high-molecular-weight allergens and a higher IgE-binding capacity than fresh fish [52]. On the other hand, it was shown in the mouse model that cooked fish extract was more allergenic than raw fish extract and also capable of triggering a parvalbumin-specific antibody response [92]. Furthermore, the heating of seafood proteins in the presence of sugars leads to the formation of advanced glycation end products (Maillard reaction), the so-called advanced glycation end products (AGEs) [17, 32, 75]. These AGEs are able to stimulate the absorption of allergens by antigen-presenting cells so that heated seafood allergens may be more potent than their non-heated counterparts [24, 33]. 

## Diagnostics 

The diagnosis of a seafood allergy is based on medical history, allergy tests and, if necessary, challenge tests (oral or by inhalation) (double-blind, placebo-controlled if possible) [10, 29, 67]. Based on the patient’s history, specific IgE serum antibodies are determined in vitro, and rubbing and/or prick tests are performed in vivo [42, 86, 87]. The basophil activation test can be useful as an additional in vitro test [80]. However, this test has not yet been established in clinical routine because of lack of clinical validation [10, 33]. Challenge testing is not necessary if medical history, medical documentation, and allergy test results are clearly correlated, and the patient is symptom-free during seafood avoidance (e.g., diagnostic elimination diet [100]), [11, 66]. Important to note, none of the allergy tests can reliably predict the risk of life-threatening anaphylaxis to seafood. 

Since the allergens of closely related seafood are extremely similar, it is generally useful to test selected representatives of different taxonomic families. For some seafood allergic persons, it is also of great interest to know to which taxonomic species they are allergic to and which ones they tolerate in order to adjust their allergen avoidance and, if necessary, dietary habits accordingly [42]. 

### Specific IgE determination 

Allergen extracts and components from different kinds of seafood are commercially available for specific IgE determination in serum [42]. The allergen extracts are a complex protein mixture of allergenic and non-allergenic seafood components, with variable composition [27, 38, 48]. Some seafood allergens may even be completely absent from the difficult-to-standardize extracts, causing a diagnostic gap, i.e., false negative test results [27, 86, 87]. 

Modern approaches to improving the diagnosis of seafood allergies aim to use the actual allergenic components instead of allergen extracts [2, 33, 42, 86, 87]. Using recombinant DNA technology, it is possible to produce recombinant allergens that are pure, precisely defined, and characterized. Individual allergen components are already commercially available such as the fish parvalbumins of carp rCyp c 1 and cod rGad c 1 and the shrimp tropomyosin rPen a 1 [36, 42, 100]. Nevertheless, the use of allergen extracts remains indispensable for the time being, since quite a few (up to 1/3 [87]) of those persons allergic to seafood are sensitized to other, mostly minor allergens [38, 45, 100]. The commercial availability of further, not only major but also minor, allergen components for specific IgE determination therefore represents an important perspective for a more accurate in vitro diagnosis. Based on clinical studies, a diagnostic test consisting of a combination of seafood allergen components will have to be defined [38, 86]. 

### Prick test 

Since allergy test solutions have to be approved as pharmaceuticals due to European legislation, the panel of commercially available seafood allergen extracts for prick testing is limited. Mostly only the prick-to-prick test with fresh or processed native material remains [15, 20, 33, 42, 82, 100]. The prick-to-prick test with native seafood is considered more sensitive than the prick test with commercially available allergen extracts [61], especially if the seafood is prepared with the same processing method as when the allergic reaction was triggered [97]. In addition, commercially available extracts from different manufacturers are characterized by very variable allergen contents, which is mainly due to the different preparation methods of the test solutions [68]. 

## Occupational occurrence 

In Germany, allergologically relevant contact with seafood allergens has to be considered for professionals in each of the following occupational groups and subgroups (quarterly number of employees as of September 30, 2019; Statistics of the Federal Employment Agency, Nuremberg, Germany, April 2020 [81]):

- Fishing industry (2,954 employees) including fish farming and fishing
- Fish processing (2,781 employees) in the area of food and luxury food production
- Food preparation (741,078 employees) including cooks
- Food control (4,557 employees)
- Food sale (487,597 employees).

According to the employment statistics of the Federal Employment Agency, a total of 41,742,042 people in Germany were registered for social security and marginal part-time employment as of September 30, 2019 [81], which indicates a 2.9% share of potentially work-related seafood exposure in Germany. 

Occupational seafood allergies are of great importance in the fishing and fish-processing industries [21, 82]. Inhalation is the most common route of exposure. The allergens enter the respiratory tract as aerosolized particles, vapors, or dust during work processes such as cleaning, cutting, cooking, or drying of seafood [33]. For example, 30 ng/m^3^ aerosolized fish antigen is sufficient to cause sensitization and workplace-related asthmatic reactions [75, 82]. Respiratory allergies among (deep-sea) fishers, fish, crab and shrimp processors, mussel openers, seafood vendors, and fish traders/sellers have been documented [31, 33]. 

In the field of food preparation, cooks are among the most exposed professional subgroups. For instance, their tasks include filleting fresh fish. During preparation, they may inhale aerosolized seafood particles [56, 84]. Finally, they have to season dishes containing seafood. In addition to a case series [12], there are several case reports on allergies caused by seafood in professional chefs [1, 31, 33, 62, 64, 71]. 

## Therapy 

Up to now, there is no causal therapy for seafood allergy [29, 42, 67]. Patients with seafood allergy suffer from their symptoms for the rest of their lives [11, 19, 29, 53, 54, 67, 100], even after the end of work-related exposure [34]. They must therefore be informed in detail about the possible consequences of continuous exposure to seafood [55], especially because of a constant threat of life-threatening anaphylaxis [73, 74]. Since the severity of a previous systemic allergic reaction cannot predict the severity of a subsequent one, they should be provided with an emergency kit – containing dimetindene maleate drops, betamethasone solution, and two epinephrine auto-injectors as well as for asthmatics, possibly also salbutamol aerosol – and information and training on their use [7, 10, 11, 12, 19, 37, 40, 69, 74, 79, 82, 99]. 

Specific immunotherapy as a causal therapy approach with mutated, recombinant, low-allergenic seafood proteins [29, 49, 67, 82, 84, 87, 89, 92], or specific oral tolerance induction with seafood [40] cannot be recommended at present due to the lack of standardized clinical protocols and the inherent risk of anaphylaxis [65, 67, 86, 87]. Therefore, only strict and lifelong allergen elimination diet remains. This means avoidance of the corresponding allergenic proteins – and thus, all seafood members of the same taxonomic group – which is to be followed not only at the workplace but also in private life [10, 11, 40, 42, 54, 65, 82, 83, 84, 86, 87, 89]. 

## Prevention measures 

In clinical routine, it is important to ask insured persons with work-related skin complaints on seafood exposure about respiratory complaints and vice versa. Results suggest that allergic respiratory disorders are common in those with occupational allergic contact urticaria and protein contact dermatitis, and that effective preventive measures should include skin and respiratory protection [12, 26]. In all cases, workplace/environmental as well as dietary allergen elimination strategies should be implemented [33, 42]. 

In view of the lack of generally applicable allergen workplace thresholds, the experts of the European Academy of Allergy and Clinical Immunology (EAACI; https://www.eaaci.org/, last access: 11/July/2020) consider reducing the level of allergens in the air as much as possible as the best protection [33]. For example, high exposure to aerosolized fish proteins (up to 986 ng/m^3^) including allergens has been measured during fish processing [9]. Preventive measures depend on the industry and include, for example, changes in the production process, improved room ventilation, or respiratory protection devices. 

The European Regulation on the provision of food information to consumers (Regulation (EU) No. 1169/2011 of the European Parliament and of the Council, latest consolidated version dated 12/12/2011; http://data.europa.eu/eli/reg/2011/1169/2011-12-12, last accessed: 11/July/2020) states that every packaged or bulk food product containing fish, crustaceans and mollusks, or products derived thereof must be labelled with an appropriate reference to this allergenic component. The clear intention is to support the allergic patient in everyday life [11, 42, 100]. Thus, the allergic consumer shall consult the list of ingredients at every purchase [82] so that, as far as possible, allergic reactions [95] upon unintentional consumption of seafood allergens can be prevented [75, 83]. International dishes in which fish is used as a seasoning are a good example for “hidden” allergens in food [11]. Seafood allergens can even be part of poultry or pork meat or other animal products (e.g., eggs) as a result of fattening the animals with fishmeal feed [22, 56, 83]. Increasingly, fish gelatin is being used instead of beef or pork gelatin [3, 44]. 

### Special remarks 

At the workplace of a cook, complete absence of seafood allergens must include avoiding 1) skin contact, 2) inhalation, for example during cooking, and 3) ingestion, for example during seasoning [28, 51, 69]. Bystander effects (i.e., reactions of uninvolved persons in the vicinity), which are not uncommon in the catering industry [11], should also be avoided. Hidden sources of allergens (cross-contamination during food preparation or when clearing and rinsing cookware contaminated with seafood remains [8, 95], seafood allergens in kitchen dust [51], etc.) additionally complicate the implementation. 

## Impact of an allergy 

In the case of occupational, especially systemic allergic reactions to seafood, it is difficult to safely avoid exposure to the potent allergens. In many cases, occupational reorientation is inevitable [12, 20, 33, 71]. 

As far as skin and mucosal are involved, seafood allergy with its various manifestations is defined by the clinical severity classification of the contact urticaria syndrome according to von Krogh and Maibach (Table 1) [12, 98]. The assessment procedure may include a contextual review primarily on the question of the presence of an occupational disease (BK (German für Berufskrankheit)) according to No. 5101 of the Annex to the German Ordinance on Occupational Diseases. In individual cases, the presence of a BK No. 4301 can also be determined. This is the case when the respiratory tract is involved. However, this corresponds to a uniform allergic phenotype with reactions in multiple organ systems. As such, the constellation is to be treated as a systemic disease in the case of an insured event – based on BK No. 5101 and BK No. 4301 – leading to an overall reduction in earning capacity (MdE (German for Minderung der Erwerbstätigkeit)) with respect to the impact of the allergic disorder [72, 78]. Due to the observed course of manifestation, however, respiratory diseases, such as allergic rhinopathy or allergic asthma, will rarely be objectively justified in occupational dermatology groups, since “respiratory involvement” appears to be caused primarily by swelling of the mucous membranes in the upper mouth and throat and by swelling of the tongue [12, 26]. 

The restriction to gaining employment in the general labor market due to the occupational disease is pivotal for the assessment of the MdE [14]. In addition to the proportion of closed jobs in the general labor market, the severity of the clinical manifestations of the allergic reactions is also taken into account in the assessment of the MdE. The occurrence of seafood in the general labor market provides the basis for creating or sustaining a work-related disease [14, 60]. Thus, although the occupational exposure to seafood is extremely limited and can be well defined, an allergy acquired during this occupational exposure may manifest itself not only onto the skin but also onto other organ systems as a systemic disease. 

*Impact of an allergy to seafood: mild, in justified individual cases moderate to severe*

Based on the clinical severity classification of the contact urticaria syndrome (Table 1), the following case considerations arise, which also take into account the hazard of allergen contacts outside the given work area:

1. In the case of allergic reactions manifesting on the skin and the directly adjacent mucous membranes exclusively upon work-related exposure, the *effect of an allergy* is to be assessed as “mild” due to the infrequent occurrence of seafood in allergenic form in the general labor market. If also in the case in other work areas by unexpected exposure aforementioned symptomatology occurs, then a higher estimate as “moderate” *effect of an allergy* can be justified in individual cases due to a barely foreseeable increase in the sensitization grade.
2. In the case of manifestation of a multi-organ involvement, i.e., with allergic reactions that exceed the skin organ, and which so far have only occurred in the proximate working environment, the *effect of an allergy* shall be assessed as “moderate”, considering the latent danger of a system reaction also in working areas without expected allergen contact.
3. With manifestation of a multi-organ involvement also outside of the given work area by unexpected job-related allergen exposure, the allocation of the *effect of an allergy* as “severe” is justified.

From multi-organ involvement onwards, the involvement of skin and respiratory tract organs can result in the combined presence of a BK No. 5101 and BK No. 4301 that are to be considered as a single insurance case under the aspect of a systemic disease [72, 77, 78]. 

A clinically relevant immunological cross-reaction between fish and chicken meat can have an effect on everyday life and therefore may become MdE-relevant in well-founded individual cases with otherwise often overlapping occurrence as occupational substances in the general job market. The other possible immunological cross-reactions between fish and frog or crocodile meat as well as between shellfish and mites or cockroaches are usually not relevant for MdE, but should be recorded as indirect occupational disease consequences by experts and treated at the expense of the accident insurer in individual cases of clinical relevance [77]. Since house dust mite sensitization is widespread in the general population [23], it is not usually recognized as an indirect occupational disease, unless the pre-occupational absence of corresponding clinical symptoms is comprehensible on the basis of previous medical findings. In that case, the component-specific sensitization profile can be analyzed with the currently commercially available major allergens rDer p 1, rDer p 2, and rDer p 23 as specific markers for primary sensitization as well as the minor allergen rDer p 10 as a marker for cross-reactivity between tropomyosins of invertebrates, such as crustaceans, mollusks and insects, and it shows a sole or leading sensitization to house dust mite tropomyosin (rDer p 10) [70, 88]. 

## Funding 

None. 

## Conflict of interest 

The authors state that they have no conflicts of interest with regard to the topic of this paper. 

**Table 1.** Contact urticaria syndrome, according to von Krogh and Maibach [98].

Stage	Cutaneous reactions	Extracutaneous reactions
Localized reactions		
I	– Contact urticaria – Dermatitis/Eczema – Nonspecific reactions: itching, prickling, burning	Ø
Systemic reactions		
II	– Generalized urticaria – Angioedema	Ø
III	– Urticaria in combination with	• rhinitis, conjunctivitis • asthma • orolaryngeal reactions • gastrointestinal reactions
IV	– Urticaria in combination with	• anaphylactic reactions
